# The Value of Muscle Training in Disease

**Published:** 1917-06

**Authors:** Theodore Toepel

**Affiliations:** Atlanta, Ga.


### Journal-Record of Medicine

Successor to Atlanta Medical and Surgical Journal, Established 1855
and Southern Medical Record, Established 1870

**OWNED BY THE ATLANTA MEDICAL JOURNAL COMPANY**
PUBLISHED MONTHLY

*Official Organ Fulton County Medical Society, State Examing Board, Atlanta, Birmingham and Atlantic Railroad Surgeon's Association, Chattahoochee Valley Medical and Surgical Association, Etc.*

A. W. STIRLING, M. D., C. M., D. P. H.; J. S. HURT, B. Ph., M.D.
GEO. M. NILES, M. D., W. J. LOVE, M. D., (Ala.); Associate Editors

E. W. ALLEN, Business Manager

**COLLABORATORS**

W. F. WESTMORELAND, M. D., General Surgery
F. W. McRAE, M. D., Abdominal Surgery
ALLEN H. BUNCE, Medical Societies
E. B. BLOCK, M. D., Diseases of the Nervous System
MICHAEL HOKE, M. D., Orthopedic Surgery
CYRUS W. STRICKLER, M. D., Legal Medicine and Medical Legislation
E. C. DAVIS, A. B., M. D., Obstetrics
E. G. JONES, A. B., M. D., Gynecology
ALLEN H. BUNCE, M. D., Pathology and Bacteriology
R. T. DORSEY, Jr., B. S., M. D., Medicine
L. M. GAINES, A. B., M. D., Internal Medicine
GEO. C. MIZELL, M. D., Diseases of the Stomach and Intestines
L. B. CLARKE, M. D., Pediatrics
EDGAR PAULLIN, M. D., Opsonic Medicine
THEODORE TOEPEL, M. D., Mechano Therapy
E. G. BALLENGER, M. D., Diseases of the Genito-Urinary Organs

VOL. LXIV. ATLANTA, GA., JUNE, 1917. NO. 3

### THE VALUE OF MUSCLE TRAINING IN DISEASE.

**Theodore Toepel, M.D., Atlanta, Ga.**

The assertion that muscle training is an absolute necessity to the best health cannot be made too strongly, even more so, the wider the application of the term, "muscle training." Use is the very condition of life of muscle and brain, while inactivity results either in atrophy at best, or in decay, if circumstances are worse. With discrimination all forms of muscular movements may be therapeutic; and the present intention is to point out the method of application proved most valuable by recent experience.

A short summary of physiology of muscular movements will be in place here, quoting from Du Bois-Reymond, W. Biederman, A. Mosso.

**Physiology of Muscle.**

Central Innervation of Skeletal Muscle.—Each one of the muscles of the extremities receives motor-nerve fibres from several successive nerve roots. This is most clearly seen in the case of the sterno-cleido-mastoid and the trapezius of man which are innervated by both the spinal accessory and the cervical nerves.

Fatigue of human muscles and nerves—Work, anemia, fasting, want of sleep, reduce the working power and favor the onset of fatigue. The capability of work is increased on the other hand, by rest, by the taking of food, and by massage—the latter even in case the muscle be previously not fatigued. The effect of massage after work therefore consists not only in the removal of products arising from the expenditure of energy, but also, and to a considerable extent, in the more active circulation of the blood and lymph and possibly in some alteration of metabolism thereby produced.

Fatigue of one group of muscles exercises an unmistakable influence on the other muscles, e. g., fatigue of the legs hastens fatigue of the arms; but muscle training reduces such effects.

Mosso has shown that while the mechanical work performed by a muscle decreases as fatigue comes on, the nervous effort and the intensibility of the process which call forth the contractions progressively increase. By a method especially adapted to the purpose, it may even be shown that the nervous mechanism is being greatly strained before there is any sign of the fatigue in the external work done. This, as Treves remarks, would explain the fact that athletes not infrequently are attacked by severe neurasthenic pains.

Reciprocal Relations Between the Muscles and other Organs of the Body.—In the performance of their functions the muscles influence, and in many ways are influenced by, the other organs of the body. A muscle degenerates if its connection with the central nervous system be interrupted, and within a relatively short time it becomes transformed into a mass of connective tissue. The same thing happens if the motor cells in the anterior horn of the spinal cord be destroyed by a lesion. The cause of degeneration under these circumstances is not that the muscle is inactive.

In activity, as it appears, for example, as the result of brain disease, involves a reduction of the muscle substance, an atrophy, but the muscle does not degenerate, it retains its characteristic properties. On the other hand, a muscle increases in mass by work, and there is, generally speaking, no other means of strengthening a muscle. We see therefore that a muscle receives impulses from the central nervous system which are of the greatest possible importance for the maintenance of its substance and of its natural properties.

A resting muscle has a relatively small supply of blood, but during work the quantity increases considerably owing to the widening of the blood stream produced by the action of the vasodilator nerves. Besides, we find as an accompaniment to muscular work an acceleration of the heart beat and, as a rule, an increase of arterial blood pressure. The latter is caused primarily by a contraction of blood vessels in other organs, especially those of the splanchnic region, which more than compensates for the dilatation in the muscles.

Vasodilatation in the muscles accompanying work is for the purpose of supplying them with an increased amount of oxygen and combustible materials; for a working muscle uses large quantities of oxygen and produces large quantities of carbon dioxide. In order to supply the necessary quantity of oxygen and to remove the great excess of carbon dioxide, the respiration must of course be augmented, and this should be mentioned as one of the accompaniments of muscular work.

### Therapeutic Muscle Training.

The greatest value of muscle training is in disorders arising from lack of exercise, such as muscular enfeeblement, gastric and intestinal atonies, insufficient cardiac and respiratory action, and venous congestion. To such disorders it is applicable in all periods of life, but, with the exception of the postural deformities and troubles caused by imperfect development in childhood and youth, it has its most valuable field of use in the disease of middle life. In more advanced age, too, it furnishes a method by which one may accurately prescribe measuerd quantities of exertion.

The prescription of muscle training for any individual must be founded on the bodily indications and on a study of the personal history and peculiarities in order that, while meeting and overcoming the deficiency, too much may not be demanded of muscles or nerves untrained for the work required.

### Some Specific Indications.

Lateral Curvature.—Simple lateral curvature of the spine that has not lasted long enough to induce osseous changes can in most instances be arrested, in many greatly improved, and in some can be entirely cured, by appropriate muscle training.

Infantile Paralysis.—After the acute stage has subsided it is best to begin with massage and when nothing further can be gained start the instruction in co-ordination and active muscle training. It is always best for the physician to superintend the movements in person. Each case requires separate consideration; each will tax to utmost the ingenuity, the resource, the patience of the doctor. The degree of improvement will depend on the persistence and ingenuity of the doctor, the mental capacity of the patient, and the grade of the disorder.

**Training for Deficient Function.**—It is impossible to give even an outline in a brief paper as this of the way in which deficient, backward, and imbecile children are trained to varying degrees of usefulness. It must suffice to say that the earlier stages of their teaching are wholly occupied with mechanical instruction, until they have learned, by infinitely small advances and frequent repetition, the use of muscular functions.

**Tabes Dorsalis.**—One of the most troublesome symptoms in cases of tabes dorsalis is the ataxia, or impairment of muscular coordination. This disability may reach so extreme a point as altogether to prevent the patient's walking and performing the simplest movements. By means of suitable exercises it is possible, even in bad cases, to restore in large measure the impaired co-ordination and to re-educate the patient in various movements. It need hardly be said that no influence is by these means exerted upon the lesions or upon the actual course of the disease, except in so far as the patient is made more comfortable and better able to help himself, and certain complications attendant upon the muscular inco-ordination, are removed or prevented. The treatment with this qualification, is purely symptomatic and deals only with one symptom, namely, the ataxia. The same general principles are applicable in improving the co-ordination of tabetic patients that are followed normally in mastering unaccustomed movements.

Suite 1613 Candler Building.

